# Radiomics for Predicting Prognostic Factors in Breast Cancer: Insights from Contrast-Enhanced Mammography (CEM)

**DOI:** 10.3390/jcm13216486

**Published:** 2024-10-29

**Authors:** Claudia Lucia Piccolo, Marina Sarli, Matteo Pileri, Manuela Tommasiello, Aurora Rofena, Valerio Guarrasi, Paolo Soda, Bruno Beomonte Zobel

**Affiliations:** 1 Operative Research Unit of Radiology, Fondazione Policlinico Universitario Campus Bio-Medico, Via Alvaro del Portillo 200, 00128 Rome, Italy; c.piccolo@policlinicocampus.it (C.L.P.); m.sarli@policlinicocampus.it (M.S.); m.tommasiello@policlinicocampus.it (M.T.); b.zobel@policlinicocampus.it (B.B.Z.); 2 Research Unit of Radiology, Department of Medicine and Surgery, Campus Bio-Medico University, Via Alvaro del Portillo 21, 00128 Rome, Italy; 3 Unit of Computer Systems & Bioinformatics, Department of Engineering, Campus Bio-Medico University, Via Alvaro del Portillo 21, 00128 Rome, Italy; aurora.rofena@unicampus.it (A.R.); valerio.guarrasi@unicampus.it (V.G.); p.soda@unicampus.it (P.S.)

**Keywords:** radiomics, breast cancer, contrast-enhanced mammography, prognostic factors, perilesional features, machine learning

## Abstract

**Objectives**: To evaluate the correlation between radiomic features extracted from contrast-enhanced mammography (CEM) tumor lesions and peritumoral background with prognostic factors in breast cancer (BC). **Methods**: In this retrospective, single-center study, 134 women with histologically confirmed breast cancer underwent CEM examination. Radiomic features were extracted from manually segmented lesions and lesion contours were automatically delineated using PyRadiomics. The extracted features were categorized into seven classes: First-order Features, Shape Features (2D), Gray Level Co-occurrence Matrix (GLCM), Gray Level Run Length Matrix (GLRLM), Gray Level Size Zone Matrix (GLSZM), and Neighboring Gray Tone Difference Matrix (NGTDM). Histological examination assessed tumor type, grade, receptor structure (ER, PgR, HER2), Ki67 index, and lymph node involvement. Pearson correlation and multivariate regression were applied to evaluate associations between radiomic features and prognostic factors. **Results**: Significant correlations were found between First-order Features and prognostic factors such as ER, PgR, and Ki67 (*p* < 0.05). GLCM-based texture features showed strong associations with Ki67 and HER2 (*p* < 0.01). Radiomic features from peritumoral regions, especially shape and GLSZM metrics, were significantly correlated with Ki67 and lymph node involvement. **Conclusions**: Radiomic analysis of both tumor and peritumoral regions offers significant insights into BC prognosis. These findings support the integration of radiomics into personalized diagnostic and therapeutic strategies, potentially improving clinical decision making in BC management.

## 1. Introduction

Breast cancer (BC) represents still a global health challenge, requiring ongoing innovations in diagnostics, in particular in the field of imaging, to improve early detection, precise characterization, and tailored treatment strategies.

This imperative aligns with the paradigm of delivering the right treatment to the right patient at the right time, which defines personalized medicine (PM) [1,2,3,4]. One highly promising second-level technique in breast imaging is contrast-enhanced mammography (CEM), which has the potential to offer a cost-effective alternative to contrast-enhanced breast Magnetic Resonance (MR) [5,6,7]. CEM operates on a physiopathological principle analogous to MR by investigating tumor neo-angiogenesis. In fact, through intravenous administration of organ-iodinated contrast medium, CEM is able to highlight hypervascularized areas/regions within the breast, including neoplastic lesions [8,9]. The proposed indications for CEM are mainly preoperative staging, resolution of concerns arising during mammographic and ultrasound screening, and assessment of response to neoadjuvant chemotherapy. Furthermore, CEM represents the preferred examination in specific patient groups, including those with dense breasts, individuals for whom breast MR is indicated in their conventional diagnostic pathway, and patients who have absolute or relative contraindications for MR.

In parallel with the evolution of breast imaging techniques, the field of radiomics has emerged as a promising tool in cancer detection and characterization. Radiomics involves the extraction of quantitative data from medical images, encompassing parameters related to intensity, shape, texture, and wavelength [9,10,11,12]. These data, when harnessed by machine learning (ML) and deep learning (DL) algorithms, can differentiate between malignant and benign tumors, assess cancer genetics, predict treatment responses, and contribute to comprehensive models that integrate multidisciplinary information [10,12,13,14,15,16,17]. Studies demonstrated that the convergence of personalized medicine, imaging, and radiomics heralds a transformative approach to breast cancer diagnosis and prognosis.

In the authors’ opinion, CEM’s capacity to provide cost-effective and high-sensitivity imaging with rapid acquisition times, coupled with radiomics’ quantitative insights, complement each other and hold the potential to revolutionize how we detect, classify, and predict the clinical course of breast cancer.

The objective of this study is to evaluate the correlation between radiomic features extracted from tumor lesions and peritumoral background with prognostic factors, in order to understand if the peritumoral parenchyma may have specific features that can impact on a patient’s prognosis, not detectable by human eye.

## 2. Materials and Methods

### 2.1. Study Population

This retrospective and single-center study was performed in accordance with the Declaration of Helsinki and approved by the ethics committee of our hospital.

From September 2021 to June 2023, 134 women with a histological diagnosis of breast cancer underwent contrast-enhanced mammography (CEM) at the Breast Unit of the Fondazione Policlinico Universitario Campus Bio-Medico in Rome, Italy.

The inclusion criteria were as follows: a suspicious breast lesion (BI-RADS 4 or 5) found on conventional imaging (mammography/tomosynthesis or ultrasound examination), patients being older than 18 years of age, and patients being able to perform CEM examination after signing the informed consent.

The exclusion criteria were as follows: pregnancy, iodinated contrast material allergy, renal failure, and breast prostheses.

Before the examination, renal function and coagulation parameters were evaluated and written informed consent was obtained.

### 2.2. Contrast-Enhanced Mammography (CEM)

A digital mammography unit (Senographe Pristina, GE Healthcare System^®^) was used to perform CEM examinations. In our center, CEM consists of the acquisition of the low-energy (25–29 kVp) and high-energy (45–49 kVp) images with the Dual Energy technique after two minutes from the administration of intravenous iodinated contrast medium (Omnipaque 350 mg/mL).

Before the examination, a contrast agent is administrated through an antecubital vein, preferring the contralateral arm of the target lesion. The contrast dose was usually 1.5 mL/kg body weight, at a rate of 2.5 mL/s, followed by 20 mL of saline flush automatically injected at 3 mL/s.

The two images are processed using subtraction algorithms with the production of a combined mammographic image to enable the possibility of analyzing the dynamics of enhancement of a suspected lesion, in a similar way to MRI.

The exam was performed as follows: cranio-caudal (CC) of the contralateral side, cranio-caudal (CC) of the target lesion, medio-lateral (MLO) of the target lesion, and medio-lateral (MLO) of the contralateral side.

If an enhancement was observed on the suspicious side, an additional image was taken after eight minutes in order to assess the enhancement kinetics and establish the probability of malignancy.

### 2.3. Histological Examination

Suspicious lesions classified as BI-RADS 4 or 5 on conventional imaging were biopsied using a core needle biopsy (CNB) or vacuum-assisted breast biopsy (VAAB).

Histological examination was performed by a pathologist with over 25 years of experience in breast disease, according to WHO (World Health Organization) guidelines. The histology, type and grade of the tumor and receptor structure (ER, PgR, and HER2), the Ki67 proliferation index, and the nodes’ involvement were analyzed.

The expression of ER, PR, and Ki67 were valued in percentage terms.

HER2 was considered positive (value 1) with a value of 3+, while HER2 value 1 results were considered negative (value 0). Specimens yielding an equivocal immunohistochemical result (2+) underwent an analysis by fluorescent in situ hybridization (FISH). In cases of amplification, a value of 1 (positive) was assigned to HER2; in cases of unamplified FISH, the value assigned was 0.

### 2.4. Radiomic Analysis

To extract radiomic features from both the lesions and their contours, a separate segmentation of the lesions and their contours was performed. The lesions were manually segmented using 3D Slicer software by an experienced radiologist with over 10 years of expertise in breast imaging and radiomics. Furthermore, a standardized segmentation protocol was followed to reduce variability and ensure consistency in the manual delineations. Subsequently, leveraging these segmentations, the lesion contours were automatically delineated, with parameters set to a thickness of 5 mm and a distance of 1 mm from the lesion segmentation. All segmentations were subsequently reviewed and validated by the entire research team to ensure consistency and accuracy.

### 2.5. Radiomic Feature Extraction

After the segmentation of the lesions and their contours, it was possible to extract radiomic features using an ad hoc Python script based on PyRadiomics, an open-source package designed for extracting radiomic features from medical images.

The radiomic features extracted separately from the lesions and their contours were divided into the following seven classes: First-order Features, Shape Features (2D), Gray Level Co-occurrence Matrix (GLCM) Features, Gray Level Run Length Matrix (GLRLM) Features, Gray Level Size Zone Matrix (GLSZM) Features, Neighboring Gray Tone Difference Matrix (NGTDM) Features, and Gray Level Dependence Matrix (GLDM) Features.

The First-order Features class consists of 19 statistical features that provide information about the distribution of pixel intensities within the segmented area of a medical image.

The Shape Features (2D) class is composed of 10 features that describe the geometric and morphological characteristics of regions of interest in the medical image. These features provide information about the shape, size, and spatial properties of the segmented area.

The GLCM Features class consists of 24 features used for texture analysis. They are based on the GLCM, which provides information about the spatial relationships between gray levels of pixels within the region of interest.

The GLRLM Features class is composed of 16 features based on GLRLM, which quantifies gray level runs. Gray level runs are defined as the length, in the number of pixels, of consecutive pixels that have the same gray level value. These features are useful for characterizing specific patterns and structures within segmented regions.

The GLSZM Features class consists of 16 features based on GLSZM, which quantifies gray level zones in the region of interest. These features provide information about the heterogeneity and distribution of intensity values within the segmented region.

The NGTDM Features class is composed of five features useful for capturing local texture variations based on NGTDM, which quantifies the difference between a gray value and the average gray value of its neighbors.

The GLDM Features class comprises 14 features based on GLDM, which quantifies gray level dependencies in an image. These features are helpful for analyzing the relationships between pixel intensities within a region and can be used for texture analysis.

### 2.6. Statistical Analysis

The analysis of the correlation between radiomic features and prognostic factors was performed using both univariate and multivariate approaches.

In the univariate analysis, we assessed how individual radiomic features correlate with individual prognostic factors. Specifically, for each radiomic feature–prognostic factor pair, we calculated the Pearson correlation coefficient and the *p*-value. The Pearson correlation coefficient ranges in [10,12,13,14,15,16,17], where −1 indicates a perfect negative correlation, 1 indicates a perfect positive correlation, and 0 indicates no correlation between the two variables. Results were considered statistically significant with a *p*-value less than 0.05.

In the multivariate analysis, we examined the correlation between each of the seven classes of radiomic features and individual prognostic factors to obtain a comprehensive view. This involved calculating correlations between variables using the Ordinary Least Squares (OLS) linear regression method. We treated the groups of radiomic features as independent variables and the prognostic factors as dependent variables. In particular, we considered the values of R-squared, adjusted R-squared, F-statistic, and the associated *p*-value returned by the OLS function.

R-squared is a measure of how well the independent variables in the model explain the variance in the dependent variable. It ranges in [0, 1], where 1 indicates a perfect explanation of the variance in the dependent variable, and it is used to assess the model’s fitting quality.

Adjusted R-squared is the modified form of R-squared that takes into account the number of independent variables in the model. The value of adjusted R-squared increases when we include extra variables that actually improve the model.

F-statistic is calculated by comparing the variance explained by the regression model to the variance not explained by the model. Together with the *p*-value, it determines the overall significance of the regression model.

Both univariate analysis and multivariate analysis were performed by extracting radiomic features in three distinct cases: considering only the images acquired in cranio-caudal (CC) projection, only the images acquired in medio-lateral oblique (MLO) projection, and, finally, considering all the images together.

## 3. Results

### 3.1. Univariate Analysis

#### 3.1.1. Correlation Between Features Extracted from Lesions and Prognostic Factors

In the univariate analysis, the correlation coefficients between radiomic features extracted from the lesions and prognostic factors were generally below 0.4, indicating that these features alone may not provide a strong association with the examined prognostic factors (ER, PgR, HER2). The full analysis of these correlations is available in the Supplementary Materials, where the coefficients for each class of radiomic features can be reviewed.

#### 3.1.2. Correlation Between Features Extracted from Lesion Contours and Prognostic Factors

In contrast, radiomic features extracted from the lesion contours demonstrated stronger correlations with prognostic factors, with several Pearson coefficients exceeding 0.4. Specifically, Uniformity (ER, r = 0.4719, *p* = 0.0009), Minimum (PgR, r = 0.4207, *p* = 0.0036), Gray Level Non-uniformity Normalized (ER, r = 0.4226, *p* = 0.0034; PgR, r = 0.4792, *p* = 0.0008), Gray Level Variance (HER2, r = 0.4096, *p* = 0.0047), Size Zone Non-uniformity (HER2, r = 0.4277, *p* = 0.0030), and Strength (HER2, r = 0.5683, *p* = 0.0000) exhibited significant positive correlations. These findings suggest that features from the lesion contours may provide more clinically relevant information for predicting prognostic factors compared to those extracted from the lesions themselves. This highlights the potential clinical implications of contour analysis in offering additional insights into tumor biology and enhancing clinical management strategies. A summary of the radiomic features with high Pearson correlation coefficients (≥0.4) is presented in Table 1, while the full set of correlation results across all feature classes can be found in Tables S1–S17 in the Supplementary Materials.

### 3.2. Multivariate Analysis

#### 3.2.1. Correlation Between Feature Classes Extracted from Lesions and Prognostic Factors

**Figure 1:**
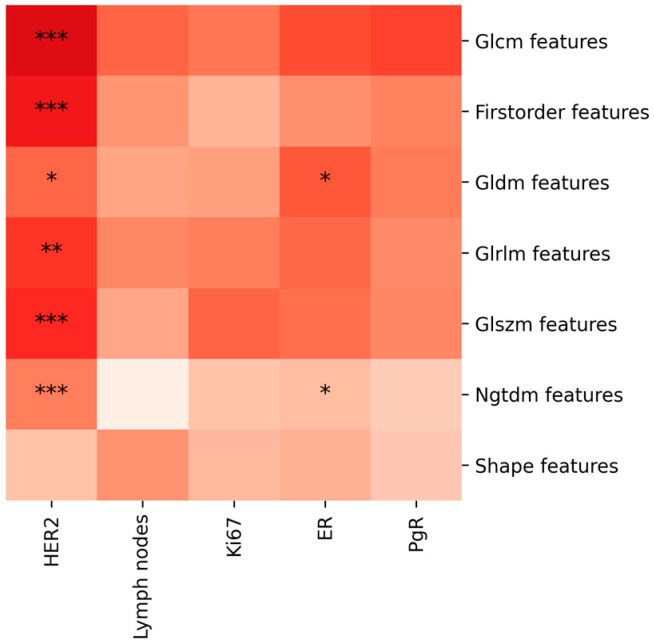
The diagram shows the multivariate analysis results examining the correlation between features extracted from lesion contour segmentation and prognostic factors, evaluated on the CC projection. The asterisks denote levels of statistical significance (*p* < 0.05, *p* < 0.01, *p* < 0.001), and the intensity of the color indicates the strength of the correlation, with darker shades representing stronger correlations.

**Figure 2:**
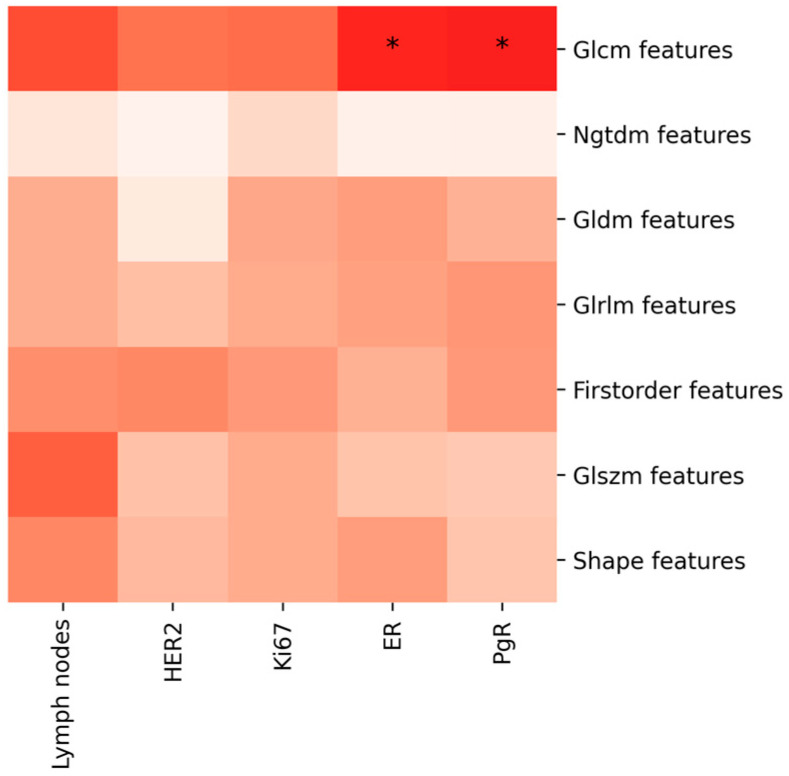
The diagram shows the multivariate analysis results examining the correlation between features extracted from lesion contour segmentation and prognostic factors, evaluated on the MLO projection. The asterisks denote levels of statistical significance (*p* < 0.05, *p* < 0.01, *p* < 0.001), and the intensity of the color indicates the strength of the correlation, with darker shades representing stronger correlations.

**Figure 3:**
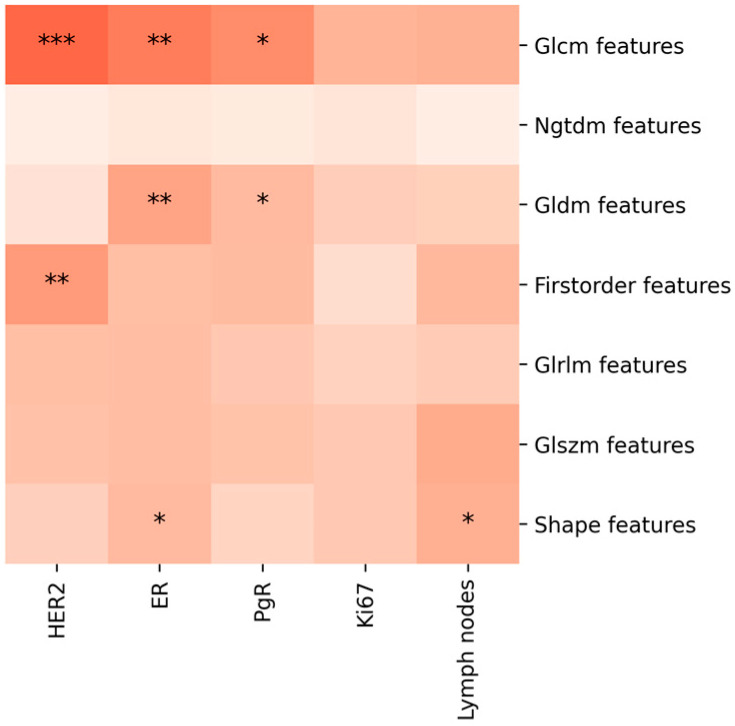
The diagram shows the multivariate analysis results examining the correlation between features extracted from lesion contour segmentation and prognostic factors, evaluated on both the CC and MLO projections. The asterisks denote levels of statistical significance (*p* < 0.05, *p* < 0.01, *p* < 0.001), and the intensity of the color indicates the strength of the correlation, with darker shades representing stronger correlations.

To further explore the relationship between radiomic features and prognostic factors, we conducted a multivariate analysis to account for the combined effect of multiple feature classes. When we extract radiomic features from lesions, the multivariate analysis reveals that the correlation between the seven feature classes and prognostic factors is more robust from a statistical perspective when we take into account the features extracted from both CC and MLO images, rather than from either CC or MLO images alone. Specifically, if we focus on the feature classes extracted solely from MLO images, there are no statistically significant correlations with prognostic factors. When we focus on the feature classes extracted solely from CC images, statistically significant correlations with prognostic factors (*p*-value < 0.05) are observed only for the combinations of feature classes and prognostic factors such as GLCM-ER, GLCM-PgR, GLRLM-ER, and NGTDM-ER (Figure 1, Figure 2 and Figure 3).

In contrast, when considering the features extracted from both CC and MLO images, the pairs of feature classes and prognostic factors that exhibit statistically significant correlations increase. The relevant pairs and their corresponding values of R2, adjusted R2, F-statistic, and *p*-value are presented in Table 2.

#### 3.2.2. Correlation Between Feature Classes Extracted from Lesion Contours and Prognostic Factors

In line with the findings from the univariate analysis, it is important to note that when radiomic features are derived from lesion contours, the correlation between the feature classes and prognostic factors is statistically significant not only when the features are extracted from the set of CC and MLO images but also when extracted from the CC images alone. Specifically, in this case all feature classes, except for the Shape Features class, exhibit statistically significant correlations with the HER2 prognostic factor. Notably, for four of these feature classes the *p*-value is less than 0.0001.

Table 3, Table 4 and Table 5 present the pairs of feature classes and prognostic factors that exhibit statistically significant correlations when the feature classes are extracted from the lesion contours in the set of CC and MLO images, in CC images only, and in MLO images only.

These correlations suggest that radiomics could complement traditional diagnostics, helping to better predict tumor behavior and guide personalized treatment, especially when conventional methods are limited.

## 4. Discussion

The potential of radiomics applied to breast imaging has been investigated recently, and studies have already demonstrated the additive value of radiomics on MRI in breast cancer evaluation and prognosis [10,11,12,13,14,15,16,18]. A recent systematic review by Vasselli et al. highlights that only a few articles in the literature investigated the association between CEM imaging radiomics features and prognostic factors, suggesting that the use of this technique in cancer prognosis and monitoring is still to be deeply investigated [16]. In this context, our study sought to elucidate the relationship between radiomics features from tumor lesions and their contours, and the prognostic factors in breast cancer. The use of both univariate and multivariate analyses has provided a more comprehensive understanding of the diagnostic potential of CEM radiomics. The univariate analysis demonstrated statistically significant relationships between specific radiomic features of the lesion and prognostic factors. Notably, First-order Features, such as Mean Absolute Deviation, Minimum, and Uniformity, exhibited strong correlations with ER, PgR, Ki67, and HER2, indicating their potential as reliable indicators of tumor characteristics and behavior in breast cancer. More specifically, Mean Absolute Deviation and Minimum displayed a significant correlation with hormonal receptors ER and PgR, suggesting that internal pixel intensity variation within tumor lesions and the minimum intensity value might serve as indirect indicators of hormonal activity within the tumor. Uniformity, on the other hand, showed a strong correlation with proliferation marker Ki67 and HER2 status, with particular emphasis on its association with Ki67, as it essentially measures the consistency of pixel intensities, hinting at the possibility that more uniform tumors may present a different proliferation profile. The texture analysis, particularly the GLCM, GLDM, GLRLM, and GLSZM features, further revealed a complex relationship with prognostic factors. These features, which characterize the texture and inter-pixel relationships, provide an intricate view of the tumor’s internal structure, potentially mirroring its biological behavior. These results are consistent with recent findings in the literature, emphasizing the role of texture features in reflecting tumor biology [17,19,20]. The analysis of Shape Features (2D), specifically the Maximum 2D Diameter Row and Surface Volume Ratio, revealed significant correlations with Ki67 and HER2, suggesting that these morphological and geometric characteristics of tumors could be indicators of tumor aggressiveness and proliferative activity.

This study’s innovative approach in analyzing features extracted from the lesion contours unveiled new diagnostic possibilities. These features, when extracted from both CC and MLO images, particularly First-order Features, showed significant correlations with ER and PgR. The texture analysis, particularly the GLCM, from both CC and MLO images and only CC images, confirms also for the perilesional area a strong statistically significant correlation with prognostic factors (ER, PgR, and Ki67). The analysis of Shape Features (2D) in the peritumoral area from CC and MLO images offers significant clinical implications as it correlates with Ki67 and lymph node involvement, suggesting that the geometric characteristics of the peripheral region of the tumor are indicative of its biological behavior, including proliferation rates and metastatic potential. This finding underscores the importance of considering not just the tumor itself but also its surrounding environment in radiomic analyses, highlighting the potential for more nuanced and comprehensive assessments in breast cancer diagnosis and treatment planning. The multivariate analysis of radiomic features extracted from breast lesions reveals critical insights with significant clinical implications. When features are derived from both CC and MLO images, the statistical robustness of correlations with prognostic factors is markedly enhanced compared to using either view alone. This comprehensive approach highlights the importance of a holistic analysis in breast cancer imaging, as it captures a more complete representation of the tumor’s characteristics, as also suggested by Zhang et al. [19,20,21]. For instance, when analyzing features solely from MLO images, no significant correlations with prognostic factors are observed. However, when considering features from CC images, certain correlations emerge, such as GLCM with ER and PgR, and NGTDM with ER. These findings suggest that while individual image views provide valuable information, they might not fully capture the tumor’s complexity. The scenario changes significantly when combining data from both CC and MLO images. This approach reveals a broader range of statistically significant correlations across various feature classes and prognostic factors, such as First-order Features with ER, PgR, and lymph nodes, and GLCM features with ER, PgR, HER2, and lymph nodes. These correlations are supported by higher R2 values, indicating a stronger predictive power. Furthermore, when radiomic features are extracted from lesion contours, the analysis yields significant correlations, particularly with the HER2 prognostic factor, even when considering CC images alone. This finding highlights the potential of contour-based radiomic analysis in predicting HER2 status.

The implications of these findings for clinical practice are significant. They suggest that a more comprehensive radiomic analysis, incorporating data from multiple image views and considering both the lesion and its contours, can provide a more accurate and detailed understanding of the tumor’s biological behavior. This could lead to more personalized treatment strategies, improving patient outcomes by tailoring therapies to the specific characteristics of each tumor.

The main limitations of this study include the relatively small cohort size and the use of manual segmentation, which may reduce the generalizability and reproducibility of the findings. Although the segmentation was performed by an experienced radiologist using a standardized protocol and reviewed by all members of the research team, manual methods are inherently prone to variability. Future studies could benefit from using automated segmentation techniques to improve consistency. Additionally, globally standardized segmentation methods could further enhance reproducibility across studies. Larger and more diverse patient populations are also needed to validate the robustness of the radiomic features identified in this analysis and ensure broader applicability.

## 5. Conclusions

This study represents a significant advancement in breast cancer radiomics, providing a systematic analysis of radiomic features from both tumor lesions and their contours. The results underscore the potential of radiomic analysis as a tool not only for diagnosis but also for tailoring treatment strategies to individual patients. Notably, this study demonstrates how radiomic features can be used to extract clinically relevant information from imaging data, potentially aiding in more accurate risk stratification and treatment planning.

Overall, this study highlights the need for an integrated, multi-dimensional approach to radiomic analysis to maximize its utility in breast cancer diagnosis and treatment planning. Incorporating radiomic features into clinical practice could enable more personalized treatment strategies tailored to each tumor’s biology, supporting more precise therapeutic decisions and improved patient outcomes.

## Figures and Tables

**Table 1:** Pearson correlation coefficients (≥0.4) between selected radiomic features and prognostic factors. This table highlights the most significant positive correlations observed in the univariate analysis. The radiomic features with coefficients ≥ 0.4 are reported, showing their association with key prognostic factors such as ER, PgR, and HER2. The corresponding *p*-values are provided to indicate statistical significance.

Radiomics	Prognostic	Pearson’s Coefficient	*p*-Value
Uniformity	ER	0.4719	0.0009
Minimum	PgR	0.4207	0.0036
Gray Level Non-uniformity Normalized	ER	0.4226	0.0034
Gray Level Non-uniformity Normalized	PgR	0.4792	0.0008
Gray Level Variance	HER2	0.4096	0.0047
Size Zone Non-uniformity	HER2	0.4277	0.0030
Strength	HER2	0.5683	0.0000

**Table 2:** Correlation between feature classes extracted from lesions and prognostic factors.

Radiomics	Prognostic	R2	R2 Adjusted	F-Statistic	*p*-Value
First-order Features	ER	0.3372	0.1939	2.3529	0.0070
First-order Features	PgR	0.2878	0.1338	1.8687	0.0375
First-order Features	Lymph nodes	0.3377	0.1667	1.9755	0.0294
GLCM Features	ER	0.4121	0.2219	2.1668	0.0081
GLCM Features	PgR	0.3876	0.1895	1.9565	0.0187
GLCM Features	HER2	0.4461	0.2669	2.4890	0.0022
GLCM Features	Lymph nodes	0.4688	0.2602	2.2468	0.0077
GLRLM Features	ER	0.3043	0.1539	2.0233	0.0222
GLRLM Features	Ki67	0.2793	0.1235	1.7922	0.0484
GLRLM Features	HER2	0.3265	0.1809	2.2425	0.0104
GLRLM Features	Lymph nodes	0.3858	0.2273	2.4340	0.0065
GLSZM Features	Ki67	0.3570	0.2179	2.5674	0.0033
NGTDM Features	ER	0.1637	0.1145	3.3267	0.0086
Shape Features	Ki67	0.2314	0.1243	2.1616	0.0249
Shape Features	Lymph nodes	0.3228	0.2116	2.9030	0.0035

**Table 3:** Correlation between feature classes and prognostic factors when feature classes are extracted from the contours of the lesions in the set of CC and MLO images.

Radiomics	Prognostic	R2	R2 Adjusted	F-Statistic	*p*-Value
First-order Features	HER2	0.3547	0.2256	2.7487	0.0021
GLCM Features	ER	0.4447	0.2651	2.4760	0.0023
GLCM Features	PgR	0.3956	0.2000	2.0232	0.0144
GLCM Features	HER2	0.5042	0.3438	3.1437	0.0002
GLDM Features	ER	0.3281	0.2043	2.6511	0.0034
GLDM Features	PgR	0.2565	0.1195	1.8729	0.0430
Shape Features	ER	0.2561	0.1525	2.4727	0.0102
Shape Features	Lymph nodes	0.2918	0.1755	2.5102	0.0104

**Table 4:** Correlation between feature classes and prognostic factors when feature classes are extracted from the contours of the lesions in the set of CC images.

Radiomics	Prognostic	R2	R2 Adjusted	F-Statistic	*p*-Value
First-order Features	HER2	0.7317	0.5976	5.4556	0.0000
GLCM Features	HER2	0.7936	0.5963	4.0208	0.0008
GLDM Features	ER	0.5498	0.3465	2.7043	0.0103
GLDM Features	HER2	0.5115	0.2909	2.3188	0.0252
GLRLM Features	HER2	0.6345	0.4328	3.1463	0.0036
GLSZM Features	HER2	0.6811	0.5051	3.8708	0.0008
NGTDM Features	ER	0.2489	0.1551	2.6517	0.0367
NGTDM Features	HER2	0.4399	0.3699	6.2831	0.0002

**Table 5:** Correlation between feature classes and prognostic factors when feature classes are extracted from the contours of the lesions in the set of MLO images.

Radiomics	Prognostic	R2	R2 Adjusted	F-Statistic	*p*-Value
GLCM Features	ER	0.6892	0.3784	2.2178	0.0341
GLCM Features	PgR	0.7037	0.4074	2.3754	0.0241

## Data Availability

Data are unavailable because of the privacy.

## Supplementary Materials

The following supporting information can be downloaded at: https://www.mdpi.com/article/10.3390/jcm13216486/s1, Tables S1–S17: The full set of correlation results across all feature classes.
