# Supplementary material for: Radiomics for Predicting Prognostic Factors in Breast Cancer: Insights from Contrast-Enhanced Mammography (CEM)
Source: J Clin Med. 2024 Oct 29;13(21):6486. doi: 10.3390/jcm13216486 (PMC11546631; doi:10.3390/jcm13216486)
Supplement: Supplementary file 1 [file jcm-13-06486-s001.zip › jcm-3248949-supplementary.pdf]

Supplementary material

Table S1. Correlation between First Order features extracted from lesions and prognostic factors.

Radiomics	Prognostic	Pearson's coefficient	p-value
Mean Absolute Deviation	ER	-0,2706	0,0095
Mean Absolute Deviation	PgR	-0,2430	0,0203
Minimum	ER	0,2724	0,0090
Minimum	PgR	0,2113	0,0444
Uniformity	Ki67	-0,3232	0,0018
Uniformity	HER2	-0,2245	0,0324

Table S2. Correlation between GLCM features extracted from lesions and prognostic factors.

Radiomics	Prognostic	Pearson's coefficient	p-value
Difference Average	ER	-0,271	0,009
Difference Average	PgR	-0,22765	0,029988
Difference Entropy	ER	-0,28536	0,006111
Difference Entropy	Ki67	0,266948	0,01053
Id	ER	0,274892	0,008363
Id	Ki67	-0,27539	0,008241
Idm	ER	0,282278	0,00671
Idm	Ki67	-0,27131	0,009287
Joint Average	ER	-0,28433	0,006305
Joint Average	PgR	-0,21911	0,036918
Joint Energy	Ki67	-0,34001	0,000976
Joint Energy	HER2	-0,22403	0,032777
Maximum Probability	Ki67	-0,31131	0,002668
Maximum Probability	HER2	-0,2435	0,020025
Sum Average	ER	-0,28433	0,006305
Sum Average	PgR	-0,21911	0,036918

Table S3. Correlation between GLDM features extracted from lesions and prognostic factors.

Radiomics	Prognostic	Pearson's coefficient	p-value
Dependence Variance	ER	-0,2882	0,0056
Dependence Variance	PgR	-0,2151	0,0406
Dependence Variance	Ki67	0,2271	0,0304
Small Dependence Emphasis	ER	-0,2831	0,0065
Small DependenceEmphasis	PgR	-0,2277	0,0300
Small Dependence Emphasis	Ki67	0,2244	0,0325

Table S4. Correlation between GLRLM features extracted from lesions and prognostic factors.

Radiomics	Prognostic	Pearson's coefficient	p-value
Gray Level Non Uniformity Normalized	Ki67	-0,3658	0,0004
Gray Level Non Uniformity Normalized	HER2	-0,2424	0,0206
Long Run High Gray Level Emphasis	ER	-0,2702	0,0096
Long Run High Gray Level Emphasis	PgR	-0,2160	0,0398
Run Length Non Uniformity Normalized	ER	-0,3286	0,0015
Run Length Non Uniformity Normalized	PgR	-0,2291	0,0289

Short Run Emphasis	ER	-0,3219	0,0019
Short Run Emphasis	PgR	-0,2595	0,0130

Table S5. Correlation between GLSZM features extracted from lesions and prognostic factors.

Radiomics	Prognostic	Pearson's coefficient	p-value
Zone Entropy	Ki67	0,3551	0,0006
Zone Entropy	HER2	0,2209	0,0354
Zone Percentage	ER	-0,2983	0,0041
Zone Percentage	PgR	-0,2584	0,0134
Zone Percentage	Ki67	0,2198	0,0364

Table S6. Correlation between Shape Features (2D) extracted from lesions and prognostic factors.

Radiomics	Prognostic	Pearson's coefficient	p-value
Maximum 2D Diameter Row	Ki67	0,24803	0,01776
Maximum 2D Diameter Row	Lymph nodes	0,36888	0,00082
Surface Volume Ratio	Ki67	-0,29765	0,00416
Surface Volume Ratio	HER2	-0,27684	0,00790

Table S7. Correlation between First Order features extracted from CC and MLO images and prognostic factors.

Radiomics	Prognostic	Pearson's coefficient	p-value
90th Percentile	ER	-0,3556	0,0005
90th Percentile	PgR	-0,2749	0,0084
Entropy	ER	-0,3525	0,0006
Entropy	PgR	-0,3523	0,0006
Uniformity	ER	0,3440	0,0008
Uniformity	PgR	0,2888	0,0055

Table S8. Correlation between First Order features extracted from CC images and prognostic factors.

Radiomics	Prognostic	Pearson's coefficient	p-value
90th Percentile	ER	-0,4537	0,0015
90th Percentile	PgR	-0,4014	0,0057
Entropy	ER	-0,5066	0,0003
Entropy	PgR	-0,4515	0,0016
Mean Absolute Deviation	ER	-0,3778	0,0096
Mean Absolute Deviation	PgR	-0,3106	0,0356
Minimum	ER	0,3472	0,0181
Minimum	PgR	0,4207	0,0036
Range	ER	-0,3573	0,0148
Range	PgR	-0,3818	0,0088
Range	HER2	0,3236	0,0283
Uniformity	ER	0,4719	0,0009
Uniformity	PgR	0,3548	0,0155
Variance	ER	-0,3765	0,0099
Variance	PgR	-0,3067	0,0382

Table S9. Correlation between GLCM features extracted from CC and MLO images and prognostic factors.

Radiomics	Prognostic	Pearson's coefficient	p-value
Contrast	ER	-0,3441	0,0008
Contrast	PgR	-0,2650	0,0111
Difference Average	ER	-0,2828	0,0066
Difference Average	PgR	-0,2123	0,0434
Difference Variance	ER	-0,3442	0,0008
Difference Variance	PgR	-0,2650	0,0111

Joint Energy	ER	0,3126	0,0026
Joint Energy	Ki67	-0,2237	0,0330
Joint Entropy	ER	-0,3504	0,0007
Joint Entropy	PgR	-0,3062	0,0032
Sum Entropy	ER	-0,3527	0,0006
Sum Entropy	PgR	-0,3201	0,0020

Table S10. Correlation between GLCM features extracted from CC images and prognostic factors.

Radiomics	Prognostic	Pearson's coefficient	p-value
Autocorrelation	ER	-0,3011	0,0420
Autocorrelation	PgR	-0,3874	0,0078
Cluster Prominence	ER	-0,3855	0,0082
Cluster Prominence	PgR	-0,3127	0,0344
Cluster Shade	ER	0,3874	0,0078
Cluster Shade	PgR	0,3171	0,0318
Contrast	ER	-0,4564	0,0014
Contrast	PgR	-0,3602	0,0139
Difference Average	ER	-0,4417	0,0021
Difference Average	PgR	-0,3106	0,0356
Difference Variance	ER	-0,4564	0,0014
Difference Variance	PgR	-0,3603	0,0139
Joint Average	ER	-0,3204	0,0299
Joint Average	PgR	-0,4100	0,0047
Joint Entropy	ER	-0,5199	0,0002
Joint Entropy	PgR	-0,4130	0,0043
Sum Average	ER	-0,3204	0,0299
Sum Average	PgR	-0,4100	0,0047
Sum Entropy	ER	-0,5144	0,0003
Sum Entropy	PgR	-0,4240	0,0033

Table S11. Correlation between GLDM features extracted from CC and MLO images and prognostic factors.

Radiomics	Prognostic	Pearson's coefficient	p-value
Dependence Entropy	ER	-0,3179	0,0021
Dependence Entropy	Ki67	0,2483	0,0176

Table S12. Correlation between GLCM features extracted from CC images and prognostic factors.

Radiomics	Prognostic	Pearson's coefficient	p-value
Gray Level Variance	ER	-0,37640	0,00993
Gray Level Variance	PgR	-0,30663	0,03820
High Gray Level Emphasis	ER	-0,30137	0,04182
High Gray Level Emphasis	PgR	-0,38750	0,00780
Small Dependence High Gray Level Emphasis	ER	-0,30545	0,03899
Small Dependence High Gray Level Emphasis	PgR	-0,39621	0,00641

Table S13. Correlation between GLRLM features extracted from CC and MLO images and prognostic factors.

Radiomics	Prognostic	Pearson's coefficient	p-value
Gray Level Non Uniformity Normalized	ER	0,2548	0,0148
Gray Level Non Uniformity Normalized	PgR	0,3200	0,0020
Run Length Non Uniformity	Ki67	0,3154	0,0023
Run Length Non Uniformity	Lymph nodes	0,2313	0,0403

Table S14. Correlation between GLRLM features extracted from CC images and prognostic factors.

Radiomics	Prognostic	Pearson's coefficient	p-value
Gray Level Non Uniformity Normalized	ER	0,4226	0,0034
Gray Level Non Uniformity Normalized	PgR	0,4792	0,0008
Gray Level Variance	ER	-0,4280	0,0030
Gray Level Variance	PgR	-0,4250	0,0032
High Gray Level Run Emphasis	ER	-0,3317	0,0243
High Gray Level Run Emphasis	PgR	-0,3963	0,0064
Long Run High Gray Level Emphasis	PgR	-0,4113	0,0045
Long Run High Gray Level Emphasis	HER2	0,3862	0,0080
Short Run High Gray Level Emphasis	ER	-0,3207	0,0298
Short Run High Gray Level Emphasis	PgR	-0,3935	0,0068

Table S15. Correlation between GLSZM features extracted from CC and MLO images and prognostic factors.

Radiomics	Prognostic	Pearson's coefficient	p-value
Gray Level Non Uniformity Normalized	ER	0,2948	0,0467
Gray Level Non Uniformity Normalized	PgR	0,3943	0,0067
Gray Level Non Uniformity Normalized	HER2	-0,4687	0,0010
Gray LevelVariance	PgR	-0,3433	0,0195
Gray Level Variance	HER2	0,4096	0,0047
Size Zone Non Uniformity	PgR	-0,2972	0,0449
Size Zone Non Uniformity	HER2	0,4277	0,0030
Zone Entropy	ER	-0,4331	0,0026
Zone Entropy	PgR	-0,4108	0,0046
Zone Entropy	HER2	0,3913	0,0072

Table S16. Correlation between NGTDM features extracted from CC images and prognostic factors.

Radiomics	Prognostic	Pearson's coefficient	p-value
Strength	PgR	-0,3232	0,0285
Strength	HER2	0,5683	0,0000

Table S17. Correlation between Shape features extracted from CC and MLO images and prognostic factors.

Radiomics	Prognostic	Pearson's coefficient	p-value
Maximum 2D Diameter Row	Ki67	0,2352	0,0248
Maximum 2D Diameter Row	Lymph nodes	0,3536	0,0014
